# Risk of SARS-CoV-2 Reinfection 18 Months After Primary Infection: Population-Level Observational Study

**DOI:** 10.3389/fpubh.2022.884121

**Published:** 2022-05-02

**Authors:** Maria Elena Flacco, Graziella Soldato, Cecilia Acuti Martellucci, Giuseppe Di Martino, Roberto Carota, Antonio Caponetti, Lamberto Manzoli

**Affiliations:** ^1^Department of Environmental and Prevention Sciences, University of Ferrara, Ferrara, Italy; ^2^Local Health Unit of Pescara, Pescara, Italy; ^3^Department of Medical and Surgical Sciences, University of Bologna, Bologna, Italy

**Keywords:** SARS-CoV-2, COVID-19, reinfection, Omicron variant, cohort study, Italy

## Abstract

Current data suggest that SARS-CoV-2 reinfections are rare. Uncertainties remain, however, on the duration of the natural immunity, its protection against Omicron variant, and on the impact of vaccination to reduce reinfection rates. In this retrospective cohort analysis of the entire population of an Italian region, we followed 1,293,941 subjects from the beginning of the pandemic to the current scenario of Omicron predominance (up to mid-February 2022). After an average of 277 days, we recorded 729 reinfections among 119,266 previously infected subjects (overall rate: 6.1‰), eight COVID-19-related hospitalizations (7/100,000), and two deaths. Importantly, the incidence of reinfection did not vary substantially over time: after 18–22 months from the primary infection, the reinfection rate was still 6.7‰, suggesting that protection conferred by natural immunity may last beyond 12 months. The risk of reinfection was significantly higher among females, unvaccinated subjects, and during the Omicron wave.

## Introduction

After the first documented case in August 2020 in Hong Kong (1), a number of field studies estimated the rate of SARS-CoV-2 reinfections after a primary episode (2, 3). Although the reported rates have been consistently low, the results differed according to the adopted definition of reinfection, setting, pandemic period, and follow-up duration, and uncertainties remain on the duration of the natural immunity and the impact of vaccination to decrease reinfection rates (4–6). In fact, some evidence on the degree of protection exerted by the existing vaccines against the BA.1/B.1.1.529 (Omicron) variant is available, but still preliminary (3, 7). We performed a retrospective cohort study on the entire population of an Italian region in order to estimate the incidence of SARS-CoV-2 reinfections and COVID-19 according to vaccination status, predominant viral strain, and time after primary infection.

## Methods

We included all residents in the Abruzzo Region, Italy with ≥1 positive nasopharyngeal swab detected through RT-PCR by the regional-accredited laboratories, from the start of the pandemic (March 2, 2020) up to January 4, 2022. On February 18, 2022 (to allow ≥45 days of follow-up), we extracted all data of the official vaccination (Regional database “Vaccinazioni Anti-COVID-19”), COVID-19 (“Surveillance COVID-19 Platform”), demographic (Italian “Anagrafica sanitaria”), hospital (Italian “File A - SDO”), and co-pay exemption (Italian “Esenzioni Ticket”) datasets of the National Healthcare System, merging individual information through encrypted fiscal code (8). Since the high-quality National Tax Registry has been recently used for fiscal codes generation and input in the main above databases, we used a deterministic linkage.

A reinfection was defined by the presence of two positive RT-PCR samples detected ≥45 days apart with ≥1 intermediate negative RT-PCR test (9, 10). Subjects were classified as “vaccinated” if they received ≥1 dose of BNT162b2, ChAdOx1 nCoV-19, mRNA-1273 or JNJ-78436735, ≥14 days before the reinfection. The follow-up started from the date of the first positive swab and ended the 1st day of reinfection, or was censored on February 18, 2022.

To account for some of the main potential confounders of the association between vaccination and COVID-19 or death (11), we used (a) the COVID-19 database, (b) the co-pay exemption database, and (c) the administrative discharge abstracts of the last 10 years to extract the following conditions of each resident—diabetes (ICD-9-CM codes in any diagnosis field-−250.xx); hypertension (401.xx-405.xx); major cardiovascular or cerebrovascular diseases (410.xx-412.xx; 414.xx-415.xx; 428.xx or 433.xx-436.xx); chronic obstructive pulmonary diseases—COPD (491.xx-493.xx); kidney diseases (580.xx-589.xx); cancer (140.xx-172.xx or 174.xx-208.xx).

The proportion of reinfections was computed in the total sample, and by demographic and clinical characteristics, time after primary infection (0–5, 6–11, 12–17, 18–22 months), and predominant circulating variant (pre-Omicron vs. Omicron; from December 28, 2021 on). Cox proportional hazard analysis was then used to compute the relative hazards of reinfection, after adjusting for age, gender, vaccine status (zero, one, two or more doses), severe COVID-19 after the first infection, and comorbidities, all included *a priori*. Schoenfeld's test was used to assess the validity of proportional hazards assumption. A two-sided *p*-value < 0.05 was considered significant. Stata, version 13.1 (Stata Corp., College Station, TX, 2014) was used for all analyses.

## Results

From the start of the pandemic, a total of 251,047 infections were detected among the 1,293,941 residents in the Abruzzo Region. After the exclusions of the subjects with a follow-up <45 days, or lacking negative intermediate swabs, a total of 119,266 subjects with primary infection were included in the analysis. The average time after the primary infection was 277 days (for a total of 90,557 person-years of follow-up); 3,001 subjects had a follow-up longer than 18 months (Table 1).

**Table 1:** Number of SARS-CoV-2 reinfections by the time after first infection.

	**Total sample**	**Reinfections^a^**	
	**(*n* = 119,266)**	**(*n* = 729)**	
Mean time after the primary infection, days (SD)	277 (179)	349 (114)	–
Months since first infection	*N*	Rate/1,000 (*n*)	95% CI (%)
Less than 6 (<183 days)	44,541	1.8 (82)	(0.15–0.23)
6–11 (183–364 days)	27,146	10.7 (291)	(0.95–1.20)
12–17 (365–546 days)	44,578	7.5 (336)	(0.68–0.84)
18–22 (547–684 days)	3,001	6.7 (20)	(0.41–1.03)

a *Two positive RT-PCR samples detected ≥45 days apart with ≥1 negative RT-PCR test collected between the first and second episodes*.

*CI, confidence interval*.

Overall, the incidence of reinfection was 6.1‰ (n = 729). Eight subjects were hospitalized due to COVID-19 (8/119,266: 7/100,000; none among the youngest), and two died (a 73-year old female, with major cardiovascular disease and diabetes, and a 79-year old male, with cancer, COPD, and kidney disease).

As shown in Table 2, the reinfection rate was significantly higher among females, younger subjects, and unvaccinated individuals (11.2 vs. 3.5‰ among those who received ≥2 vaccine doses). Moreover, a markedly higher rate of reinfections was recorded during the first 54 days of the Omicron wave (*n* = 613; 11.4 per day) than during the 317 days of the pre-Omicron period (*n* = 116; 0.4 per day). In contrast, the incidence of reinfection did not vary substantially by baseline comorbidities and over time: after 18 or more months from the primary infection (up to 22 months), the reinfection rate was still 6.7‰. The multivariable analysis (the Cox proportional hazards model adjusted for all the recorded variables, included *a priori*) confirmed univariate results (Table 2). When the analysis was stratified by circulating variant, the effectiveness of ≥2 vaccine doses was slightly higher in pre-Omicron (adjusted hazard ratio—HR vs. unvaccinated: 0.20; 95% confidence interval: 0.12–0.33) than the Omicron wave (HR: 0.28; 0.23–0.35 – data not shown). Although the incidence of reinfection was lower among those with a history of severe COVID-19 manifestation, the numbers were too scarce to detect a significant difference at multivariable analysis.

**Table 2:** SARS-CoV-2 reinfections, overall and by selected covariates.

	**Total sample**	**Reinfections^a^**		**Reinfection**
**Variables**	***N***	**Rate/1,000 (*n*)**	***p*-value^b^**	**Adj. HR (95% CI)**
Overall	119,266	6.1 (729)	–	–
Age class, years			<0.001	
60+	24,989	3.3 (83)		1 (ref. cat.)
30–59	54,046	6.2 (335)		2.14 (1.61–2.86)
0–29	40,231	7.7 (311)		2.00 (1.53–2.62)
Mean age in years (SD)	41.6 ± 21.9	35.2 ± 21.6	<0.001	0.99 (0.99–1.00)
Gender			<0.001	
Males	58,783	5.3 (312)		1 (ref. cat.)
Females	60,483	6.9 (417)		1.32 (1.14–1.53)
Severe COVID-19 after the first infection
No	117,790	6.2 (726)	0.043	1 (ref. cat.)
Yes	1,476	2.0 (3)		0.35 (0.11–1.09)
Diabetes^c^			0.4	
No	114,224	6.2 (703)		1 (ref. cat.)
Yes	5,042	5.2 (26)		1.36 (0.89–2.08)
Hypertension^c^			0.002	
No	108,097	6.3 (685)		1 (ref. cat.)
Yes	11,069	3.9 (44)		0.98 (0.68–1.40)
Cardiovascular diseases^c^			0.001	
No	109,350	6.3 (692)		1 (ref. cat.)
Yes	9,916	3.7 (37)		0.76 (0.52–1.11)
COPD^c^			0.7	
No	115,548	6.1 (708)		1 (ref. cat.)
Yes	3,718	5.6 (21)		1.02 (0.66–1.59)
Kidney diseases^c^			0.008	
No	117,749	6.2 (725)		1 (ref. cat.)
Yes	1,517	2.6 (4)		0.49 (0.18–1.33)
Cancer^c^			0.062	
No	115,147	6.2 (713)		1 (ref. cat.)
Yes	4,119	3.9 (16)		0.75 (0.45–1.24)
COVID-19 Vaccination^d^			<0.001	
No	30,690	11.2 (343)		1 (ref. cat.)
1 dose	36,679	5.6 (204)		0.31 (0.26–0.38)
2 or 3 doses	51,897	3.5 (182)		0.27 (0.22–0.32)

*Adj., adjusted; HR, hazard ratio; CI, confidence interval; SD, standard deviation; ref. cat., reference category; NE, not estimable*.

a *Two positive RT-PCR samples detected ≥45 days apart with ≥1 negative RT-PCR test collected between the first and second episodes*.

b *Chi-squared test*.

c *Subjects with the selected comorbidities in the Regional co-pay exemption database (the Italian “Esenzioni Ticket” file) or a hospital admission in the last 10 years (from the Italian SDO database of administrative discharge abstracts) with the following ICD-9-CM codes in any diagnosis field-−250.xx (diabetes); 401.xx-405.xx (hypertension); 410.xx-412.xx or 414.xx-415.xx or 428.xx or 433.xx-436.xx (major cardiovascular or cerebrovascular diseases – CVD); 491.xx-493.xx (chronic obstructive pulmonary disease – COPD); 580.xx-589.xx (kidney diseases); 140.xx-172.xx or 174.xx-208.xx (cancers)*.

d *At least 14 days before the reinfection*.

## Discussion

This study confirms and expands previous findings reporting a low risk of SARS-CoV-2 reinfection, and a very low risk of severe or lethal COVID-19 for those who recovered from primary infection (3, 12, 13), suggesting that the protection conferred by the natural immunity lasts beyond 12 months. Although the marked increase of the reinfection rates during the Omicron wave is concerning, the risk of a secondary severe disease or death remained close to zero. Therefore, despite the vaccines were able to significantly reduce the likelihood of reinfection in both pre-Omicron and Omicron waves, the risk-benefit profile of multiple vaccine doses for this population should be carefully evaluated.

Interestingly, reinfection seemed to be more frequent among females, which might be a consequence of the lower attitude to routine diagnostic testing of the males (14), as well as the higher work-related exposure to the contagion of the females (15).

Our estimates might be limited by a lack of viral genotype data, which are the gold standard diagnostic method for SARS-CoV-2 reinfections (5). Also, to reduce the likelihood of defining as reinfected the subjects with prolonged viral shedding, we followed CDC criteria (9) and classified as reinfections only the cases with ≥1 intermediate negative swab between the two positive tests. Thus, we may have missed a reinfection case if an infected subject did not have a negative swab after healing. However, according to the Italian legislation, during the pandemic, a negative swab was requested to all positive subjects to end the mandatory quarantine (16). Thus, the number of subjects who healed and did not have a negative test is likely to be low. Indeed, in this sample, only 14 subjects had two positive tests 45 days apart and no negative swab. Also, the adoption of the CDC criteria to define reinfection allowed the comparison with several previous studies on the topic (17), including two Italian field studies, which showed similar results (13, 18).

## Data Availability Statement

The raw data supporting the conclusions of this article will be made available by the authors, without undue reservation.

## Ethics Statement

The studies involving human participants were reviewed and approved by Ethics Committee of the Emilia-Romagna Region (protocol code 2525/21). The Ethics Committee waived the requirement of written informed consent for participation.

## Author Contributions

MF, GS, CAM, and LM: concept and design. GS, GDM, RC, and LM: acquisition, analysis, or interpretation of data. MF, CAM, and LM: drafting of the manuscript. GS, GDM, AC, and LM: critical revision of the manuscript for important intellectual content. MF and LM: statistical analysis. GS, GDM, RC, and AC: administrative, technical, or material support. AC and LM: supervision. LM: had full access to all the data in the study and takes responsibility for the integrity of the data and the accuracy of the data analysis. All authors contributed to the article and approved the submitted version.

## Conflict of Interest

The authors declare that the research was conducted in the absence of any commercial or financial relationships that could be construed as a potential conflict of interest.

## Publisher's Note

All claims expressed in this article are solely those of the authors and do not necessarily represent those of their affiliated organizations, or those of the publisher, the editors and the reviewers. Any product that may be evaluated in this article, or claim that may be made by its manufacturer, is not guaranteed or endorsed by the publisher.
